# Stroke survivors’ levels of community reintegration, quality of life, satisfaction with the physiotherapy services and the level of caregiver strain at community health centres within the Johannesburg area

**DOI:** 10.4102/ajod.v6i0.296

**Published:** 2017-03-30

**Authors:** Adrian Kusambiza-Kiingi, Douglas Maleka, Veronica Ntsiea

**Affiliations:** 1 Physiotherapy Department, University of the Witwatersrand, South Africa; 2 Department of Health Sciences Education, University of Limpopo, South Africa

## Abstract

**Background:**

Stroke survivors are discharged home before they are functionally independent and return home with activity limitations that would not be manageable without a caregiver.

**Aim:**

To determine stroke survivors’ levels of community reintegration, quality of life (QOL), satisfaction with the physiotherapy services and the level of caregiver strain at community health centres within the Johannesburg area.

**Method:**

This was a cross-sectional study using the following outcome measures: Maleka Stroke Community Reintegration Measure, Stroke-specific quality of life scale, Caregiver strain index and Physical therapy patient satisfaction questionnaire.

**Results:**

A total of 108 stroke survivors and 45 caregivers participated in this study. The average age of the stroke survivors was 54 years (standard deviation = 12.73) and 58% (*n* = 62) had moderate to full community reintegration. They were happy with physiotherapy services but not with parking availability and cost of services. The QOL was poor with the lowest scores for energy and highest scores for vision and language domains. Twenty five (55%) caregivers were strained. A positive correlation was found between community reintegration and satisfaction with services (*r* = 0.27, *p* < 0.0001) and QOL (*r* = 0.51, *p* < 0.0001). A negative correlation was found between community reintegration and caregiver strain (*r* = -0.37, *p* < 0.0001).

**Conclusion:**

Most stroke survivors are reintegrated into their communities except in the areas of work and education and have poor QOL and most of their caregivers are strained; however, they are satisfied with physiotherapy services.

## Introduction

There are limited statistics specifically focused on the prevalence of stroke in South Africa; however, Thorogood et al. (2007) have indicated that the prevalence is high. In a census carried out by the Southern African Stroke Prevention Initiative in 2001 in Limpopo, it was found that a crude prevalence of 300 people in every 100 000 suffer a stroke (Connor et al. 2004). It is also estimated that by 2023 there will be a 30% increase in the number of first-time stroke sufferers (Wolfe 2000). Thus, it will be necessary to know the consequences of this condition on patients and their families.

Stroke may result in motor, sensory, perceptual or cognitive deficits. These deficits, in addition to environmental and personal factors, lead to disability, hindering functional capability. Disability in the context of this study is based on the International Classification of Functioning, Disability and Health model and refers to the inability to function in multiple life areas such as walking, taking a bath, working, going to school or work, accessing social services – it is seen as a result of an interaction between a person and their environmental and personal factors (WHO 2001/2002). Motor deficits are among the most common deficits that hinder a person’s ability to complete their activities of daily living (ADLs) (Langhorne, Coupar & Pollock 2009) and can also affect the upper limb leading to poor functional use of the arm (Lo et al. 2010). This leads to problems while engaging in ADLs and community activities (Pang, Harris & Eng 2006). These limitations are not only for severe stroke because even after a mild stroke, ADLs and social roles may be affected (Rochette et al. 2007) and this may lead to participation restrictions.

In all, 39% to 65% of stroke survivors report problems with activity limitations and participation restrictions that are related to their community reintegration (Pang, Eng & Miller 2007). A Canadian study by Mayo et al. (2002) also showed that 50% of stroke survivors return to their communities to live with impairments that would not be manageable without the assistance of an able-bodied caregiver at home. This means that the patients will have limited activities because of dependence and this may result in inactivity-related deconditioning leading to decreased physical capacity (Langhammer, Lindmark & Stanghelle 2007). This is aggravated by the fact that most stroke survivors are discharged from the hospital when they still remain dependent for ADLs (Mamabolo et al. 2009). This lack of independence would also lead to lower levels of community reintegration and poor quality of life (QOL).

Despite the fact that stroke survivors still have impairments at discharge, in a study done in Washington it was shown that most of them still get discharged home with no post-discharge rehabilitation services (Edwards et al. 2006). This also contributes towards poor functional recovery and ability to engage in meaningful activities and participate in their community (Hillier & Inglis-Jassiem 2010). Thus, post-discharge rehabilitation has to be taken into consideration, more so that it is also essential to being able to participate in education, the labour market and civic life (WHO 2011), which are indicators of community reintegration. Lord and Rochester (2005), in their systematic review, established that community reintegration marks the end of their rehabilitation for many stroke survivors.

To ensure that stroke survivors achieve community reintegration, they have to be discharged with a plan for continued rehabilitation for them to reach their maximum capacity. Rehabilitation in most stroke rehabilitation settings in South Africa, including Johannesburg, can take place in various settings such as hospital outpatient department, community health centre or clinic rehabilitation department and in the patient’s home (Rhoda, Mpofu & DeWeerdt 2009). Community healthcare centres (CHCs) provide services including preventative, promotive, curative and rehabilitative care and these centres, in most cases, are the first medical point of contact for stroke survivors (Rhoda & Hendry 2003). Some patients receive private rehabilitation services. Most patients receive treatment in the government sector; however, the services are limited because of lack of resources. This is because the South African health system is inequitable, with the privileged few having disproportionate access to health services (National Health Insurance Gazette 2011).

Some stroke survivors do receive rehabilitation services at the CHCs but a clear view on their satisfaction with services provided has not emerged. The significance of patient satisfaction is that patients are more likely to adhere to exercise programmes or recommended activities when they are satisfied with the physiotherapy service (Hush, Cameron & Mackey 2011). Patient satisfaction can also be used more to measure the quality of healthcare services (Hush et al. 2011).

Literature on the levels of community reintegration of stroke survivors living in the Johannesburg areas and their satisfaction with physiotherapy services received has not been established. This led to the researchers to conduct this study with the following objectives to: (1) determine the level of community reintegration of stroke survivors in the Johannesburg areas, (2) establish the stroke survivors’ satisfaction with physiotherapy services received at the community health centres within the Johannesburg area, (3) establish the QOL of these stroke survivors, (4) establish the level of strain experienced by their caregivers, (5) establish and determine the relationship between community reintegration and caregiver strain, QOL and satisfaction with physiotherapy services.

## Method

This study was a quantitative, cross-sectional study. Stroke survivors were recruited from four community health centres. These four centres are community health centres that offered physiotherapy as part of their rehabilitation service in areas around Johannesburg at the time of the study. The sample size was based on the combined average monthly population of stroke survivors seen at these four health centres over 3 months which was 150. According to Bartlett, Kotrlik and Higgins (2001), the number of participants needed to accurately represent the views of the population under study, based on the monthly patient population, was 108. This figure was calculated at a confidence interval of 95% with reliability of 0.05 (Bartlett et al. 2001).

Stroke survivors were included if they met the following criteria: were aged more than 18 years, were with or without a caregiver, receiving physiotherapy services as an outpatient or on a home visit basis from any of the study sites, able to give verbal or written consent to take part in this study, had a stroke for more than 6 months but not more than 4 years (most improvements after stroke happen within the first 6 months and stroke survivors return to work up to 2 years after stroke) (Duff, Ntsiea & Mudzi 2014). Stroke survivors were included in this study up to 4 years post-stroke to accommodate those who had severe stroke who may have taken longer to reach a plateau of their functional level considering that some of the patients seen at the CHCs had stroke duration of more than 4 years but less than 5 years. They were excluded if they had more than one stroke, if they had receptive aphasia and if they had any other comorbidity which may affect their mobility or cognitive ability. The primary caregiver was defined as the person who spends the most time compared to any other individual in the household caring for the patient.

### Outcome measures

The Maleka Stroke Community Reintegration Measure (MSCRIM) was used to assess community reintegration. It has an urban and rural version, but for the purpose of this study, the urban version was used. This measure was found to be reliable and valid for stroke survivors in the urban townships of Johannesburg. The urban version has 40 items that are spread over the following six domains: ADLs and self-care, Social interaction and relationship, Home and family responsibilities, Social interaction, Extended family responsibilities and Work and education. The urban version of the MSCRIM is scored out of 112 by the researcher and converted to a percentage with a higher percentage score implying a higher level of community reintegration (Maleka 2010). The MSCRIM was chosen because of its high reliability coefficient of 0.95 as well as its urban version’s relation to the reality of an urban community in South Africa (Maleka 2010). It also has elements in it that relate directly to the social circumstances that were faced in the communities in the areas surrounding Johannesburg not found in other questionnaires such as attending traditional events and being able to collect water from a source outside of the participants dwelling.

The Caregiver strain index (CSI) was used to measure the subjective caregiver strain. It consists of 13 yes/no items which cover employment, finance, physical, social and time-related matters (Sullivan 2002). A total score above seven indicate that that the caregiver is strained (Robinson 1983). The CSI was chosen as most articles in the literature used it as the primary measure when investigating caregiver strain (Blake, Lincoln & Clarke 2003; Bugge, Alexander & Hagen 1999; Mudzi 2010) and thus it would be easy to compare findings considering that Mudzi’s (2010) study was conducted in Johannesburg South Africa. The CSI was found to have an internal consistency of α = 0.86 and construct validity was supported by correlations with physical and emotional health of the caregiver as well as subjective views of the caregiving situation (Sullivan 2002).

QOL was measured using the Stroke-specific quality of life scale (SSQOL), which has the following 12 domains: energy, family, roles, language, mobility, mood, personality, self-care, social roles, thinking, upper extremity function, vision and work/productivity. Higher scores indicate better function. It was chosen for its convenience of having one score, which would allow for simplified correlations between the SSQOL and MSCRIM. The SSQOL was found to be a valid and reliable measure of health-related QOL, has good internal consistency (α = 0.81–0.94), construct validity and responsiveness to change for the 12 subscales (Lin et al. 2011).

Patient satisfaction with physiotherapy services was measured using the Physical therapy patient satisfaction questionnaire (PTPSQ), which comprises 26 points as follows: first 6 asking about demographic data as well as site of injury and the other 20 questions relating to satisfaction with the physiotherapy service offered. High total scores are indicative of higher levels of patient satisfaction with the physiotherapy service they received. The PTPSQ has a Cronbach α coefficient of 0.99. The questionnaire was shown to yield reliable measurements as well as have content, construct and concurrent validity (Goldstein, Elliot & Guccione 2000). The PTPSQ was chosen for this study. Although the questionnaire was developed in America and had items that were related to bills and parking space, things not generally found at the healthcare facilities in the Johannesburg area, it was very comprehensive as well as providing a space for comments that would help enrich the discussion of this study by allowing the researcher to capture data that might not have been directly related to the question.

Demographic data sheet, which was developed just for this study, was also used to capture information such as gender, physical address, race, stroke survivor’s currents occupation, date of administration of the questionnaire, date of birth, age, marital status, whether a caregiver is present, level of education, side of the body affected by stroke and date of stroke.

### Procedure

Files at each clinic were checked regularly for stroke participants who met the criteria for inclusion in this study by the physiotherapists working at each clinic. Patients who did not meet the inclusion criteria of this study also received rehabilitation services. Participants who met inclusion criteria were contacted telephonically or through the weekly stroke groups to set appointments at the clinic. Home visits were done where the participants could not make it to the clinic. Consent was obtained from participants who met the inclusion criteria and demographic data sheet was completed by all study participants followed by the MSCRIM and SSQOL. Caregivers were assessed using the CSI. The PTPSQ was administered for each participant to determine the satisfaction with the physiotherapy services. All these were taken at once. Data collection was done as the patients who met the inclusion criteria became available until the minimum sample size was attained. Total data collection period was 12 months.

### Data analysis

Descriptive statistics were used and presented as frequencies, percentages, means and standard deviations (SDs). The data for the PTPSQ were skewed and thus median scores and interquartile ranges were calculated. The data from the MSCRIM were skewed and therefore Spearman’s coefficient was used to establish correlations between community reintegration and caregiver strain, QOL and satisfaction with physiotherapy services.

## Results

One hundred and eight participants met the inclusion criteria and they were all recruited and they all agreed to participate in this study. Forty-two percent (*n* = 45) of the 108 stroke survivors had caregivers.

### Demographic information

The mean age of stroke survivors was 54 years (SD = 12.73). The youngest participant was 20 years old and the oldest was 79 years old. Fifty-seven participants came from Alexandra township, 2 from Diepsloot, 8 from Mofolo, 19 from Hillbrow and 22 from Chiawelo (*n* = 108). The time since stroke was 26 months. Demographic information of the study participants is shown in Table 1. There were more females (56%) and most of the participants (65.7%) left high school without completing matric (final year of high school). The percentage of stroke survivors with left hemiplegia was almost equal to that of patients with right hemiplegia (53% and 47%, respectively).

**TABLE 1:** Demographic information of the stroke survivors (*n* = 108).

Participant characteristics	*n* (%)
Gender	
Male	48 (44)
Female	60 (56)
Race	
Black	100 (93)
Coloured	3 (2.7)
Asian	4 (3.7)
Other	1 (0.6)
Marital status	
Single	37 (34.3)
Married	55 (50.9)
Divorced	5 (4.6)
Widow(er)	11 (10.2)
Employment status	
Employed	32
Unemployed	59 (55)
Pensioner	31 (28)
Education	
No education	8 (7.4)
Completed primary school	8 (7.4)
High school without matric	71 (65.7)
Matriculated	15 (13.9)
Tertiary education	6 (5.6)
Affected side	
Left	57 (53)
Right	51 (47)

#### Objective 1: Stroke survivors’ level of community reintegration

Results of the level of stroke survivors’ level of community reintegration are presented in Table 2. The mean total score for the MSCRIM was 70 out of 112 (SD = 22.94). The highest score measured was 112 and the lowest score was 17. Fifty eight percent of the stroke survivors had moderate to full integration and 21% had no community integration. The MSCRIM domain scores are presented in Table 3. When viewed as a percentage of each total domain score, ‘ADL & self-care’ (36 out of 48) and then ‘Social interactions’ (9 out of 13) have the highest scores among the participants in this study. Areas that participants struggled with the most were extended family responsibilities and work and education, which both had a mean score of two out of six.

**TABLE 2:** Level of stroke survivors’ community reintegration (Maleka Stroke Community Reintegration Measure scores).

Level of integration	MSCRIM	*n* (%)
No integration	0%–40%	23 (21.3)
Minimal integration	41%–59%	23 (21.3)
Moderate integration	60%–79%	32 (29.6)
Full integration	80% and above	30 (27.8)

**TABLE 3:** Maleka Stroke Community Reintegration Measure domain scores (*n* = 108).

Domain (total domain score)	Mean ± SD
Activities of daily living and self-care (48)	36 ± 11.9
Social interaction and relationship (20)	10 ± 5.2
Home/family responsibilities and appearance (19)	9 ± 6.5
Social interactions (13)	9 ± 2.5
Extended family responsibilities (6)	2 ± 1.4
Work and education (6)	2 ± 2.3

SD, standard deviation.

#### Objective 2: Satisfaction with physiotherapy services

The mean score for patient satisfaction with physiotherapy was 92% (SD = 9.17).

The PTPSQ scores are presented in Table 4. Participants’ lowest scores were for accuracy of bills, availability of parking and cost of physiotherapy. All the other questions had full total scores.

**TABLE 4:** Physiotherapy-specific patient satisfaction questionnaire individual question scores.

Question (total score: 5 for each question)	Median (IQR)
My privacy was respected	5 (5 to 5)
Physical therapist was courteous	5 (5 to 5)
Staff members were courteous	5 (4 to 5)
Clinic scheduled appointments at convenient times	5 (4 to 5)
Satisfied with treatment by physical therapist	5 (5 to 5)
My first physiotherapy appointment was organised quickly	5 (4 to 5)
Easy to schedule visits after my first appointment	5 (4 to 5)
I was seen promptly when I arrived for treatment	5 (4 to 5)
The location of the facility was convenient for me	5 (4 to 5)
My bills were accurate	0 (0 to 0)
I was satisfied with the services offered by my physical therapist	5 (4 to 5)
Parking was available for me	0 (0 to 0)
The physiotherapist understood my condition	5 (5 to 5)
The instructions my physiotherapist gave me were helpful	5 (4 to 5)
I was satisfied with the overall quality of my physiotherapy care	5 (4 to 5)
I would recommend this facility to my family or friends	5 (5 to 5)
I would return to this facility if I required physiotherapy in the future	5 (4 to 5)
The cost of physiotherapy was reasonable	0 (0 to 0)
If I had to, I would pay for these physiotherapy services myself	5 (2.75 to 5)
Overall, I was satisfied with my experience with physiotherapy	5 (5 to 5)

IQR, Interquartile range.

#### Objective 3: Quality of life

The mean total for the SSQOL for all 108 stroke survivors in this study was 157 out of 245 (SD = 23.16) with a highest score of 235 and the lowest score of 54 out of 245. Mean scores for the SSQOL domains are presented in Table 5. Participants had problems in each of the domains with the lowest scores for the energy domain and highest scores for the vision and language domains.

**TABLE 5:** Stroke-specific quality of life scale domain scores (*n* = 108).

Domain	Mean ± SD	Score (%)
Energy (15)	8 ± 4.41	53
Family role (15)	9 ± 4.22	60
Language (25)	19 ± 6.85	76
Mobility (30)	18 ± 8.56	60
Mood (25)	15 ± 7.05	60
Personality (15)	9 ± 4.1	60
Self-care (25)	16 ± 7.74	64
Social roles (25)	14 ± 6.98	56
Thinking (15)	11 ± 3.93	73
Upper extremity function (25)	16 ± 7.34	64
Vision (15)	12 ± 3.95	80
Work/productivity (15)	9 ± 4.77	60

SD, standard deviation.

#### Objective 4: Level of strain experienced by caregivers

Twenty five (55%) caregivers had a CSI of score ≥7 meaning that they were strained and 20 (45%) had a score <7 meaning that they were not strained. The numbers of caregivers who replied ‘yes’ and ‘no’ to any of the domains are shown in Table 6. Caregivers had difficulty with the following: physical strain from taking care of the stroke survivor, having many changes to their personal plans and seeing the stroke survivors’ behavioural changes. Sleep disturbances and work adjustments were the least affected items.

**TABLE 6:** Caregiver strain index domain scores (*n* = 45).

Domain	Yes *n* (%)	No *n* (%)
Caregiver experiences sleep disturbance	10 (22)	35 (78)
It is an inconvenience	19 (42)	26 (58)
It is a physical strain	21 (47)	24 (53)
It is confining	19 (42)	26 (58)
There have been family adjustments	24 (53)	21 (47)
There have been changes in personal plans	21 (47)	24 (53)
There have been other demands on my time	20 (44)	25 (56)
There have been emotional adjustments	20 (44)	25 (56)
Some behaviour is upsetting	14 (31)	31 (69)
It is upsetting to find xxxx has changed so much from his/her former self	21 (47)	24 (53)
There have been work adjustments	10 (22)	35 (78)
It is a financial strain	17 (38)	28 (62)
Feeling completely overwhelmed	14 (31)	31 (69)

#### Objective 5: Correlation between community reintegration and caregiver strain index, stroke survivors’ quality of life and satisfaction with services

The results of correlations between MSCRIM and CSI, SSQOL and PTPSQ are presented in Table 7. There was a positive correlation between MSCRIM and SSQOL showing better QOL in participants with higher levels of reintegration. There was a tendency to get better scores between MSCRIM and PTPSQ showing a higher level of satisfaction with physiotherapy services in participants with higher levels of community reintegration. A weak negative correlation was found between the MSCRIM and CSI indicating that caregivers of participants with higher levels of integration had lower levels of strain.

**TABLE 7:** Correlation between community reintegration and Caregiver strain index, stroke survivors’ quality of life and patients’ satisfaction.

Correlation investigated	Correlation value	*p*	Summary of correlation
MSCRIM and PTPSQ	0.2745	<0.0001	Weak positive correlation
MSCRIM and SSQOL	0.5190	<0.0001	Positive correlation
MSCRIM and CSI	-0.3707	<0.0001	Weak negative correlation

CSI, Caregiver strain index; MSCRIM, Maleka Stroke Community Reintegration Measure; SSQOL, Stroke-specific quality of life; PTPSQ, Physical therapy patient satisfaction questionnaire.

## Ethical consideration

Ethical clearance was granted by the University of the Witwatersrand committee for research on human subjects: Clearance number M1404452. Participants were given an information letter that explained the procedure and both participants and caregivers were asked to complete a consent form prior to administering the questionnaires. The participants were given the option of withdrawing from the study at any time and participants remained anonymous when the findings were presented.

## Discussion

The objectives this study were to determine the level of community reintegration of stroke survivors in the Johannesburg areas, establish the stroke survivors’ satisfaction with physiotherapy services received at the community health centres within the Johannesburg area, establish the QOL of these stroke survivors and the level of strain experienced by their caregivers. The relationship between community reintegration and caregiver strain, QOL and satisfaction with physiotherapy services was also determined.

### Community reintegration after stroke

Fifty seven percent of the stroke survivors had moderate to full integration and 21% had no community integration. An explanation for those with low integration may be because of the low levels of functional ability at the time of discharge from the hospital. According to Mamabolo et al. (2009), the average stay of a survivor of stroke in Chris Hani Baragwanath, a government hospital in South Africa, is 12 days. These patients are discharged from hospital quite early and one could say that they are discharged before they reach functional independence (Mamabolo et al. 2009). To ensure early discharge leads to community reintegration, survivors of stroke need to be discharged with a plan for continued intervention in their home setting or as a rehabilitation outpatient (Mayo et al. 2000). In South Africa there is a shortage of post-discharge rehabilitation services for patients who use government facilities. The shortage of rehabilitation services was also found to be a challenge in a study by Rhoda et al. (2009) that looked at rehabilitation of stroke survivors at community health centres in the Western Cape. They established that of the 39 community health centres situated in various districts within the Western Cape, only 20 offered rehabilitation services. All the centres that offered rehabilitation services in this study had physiotherapy services, and only half offered occupational therapy services.

Reduced community reintegration post-stroke is not unique to this study. In a Hong Kong study by Pang et al. (2007), only 11% of the participants considered themselves to be reintegrated into their communities, which is much less than 28% who considered themselves fully integrated in this study. Both studies were done more than 6 months post-stroke, but in the study by Pang et al. (2007), only those above the age of 50 years were included. This may have included participants whose functional ability has deteriorated many years after their stroke because patients who are unable to maintain their activity levels post-stroke experience deterioration in their condition (Langhammer et al. 2007). Patients in the study by Pang et al. (2007) also experienced low levels of self-efficacy in their study, whereas self-efficacy or ability to manage ADLs was the one domain in this study that had the lowest complaints and this is a possible explanation for the relatively higher number of those who were reintegrated (Kluding & Gajewski 2009).

Participants experienced some difficulty in all MSCRIM domains with ADLs and self-care and social interaction showing the least amount of difficulty. In a study by Mayo et al. (2002), 77% of the participants did not struggle with basic ADLs. This finding matches the findings in this study. Participants in the study by Mayo et al. (2002) were assessed at 6 months post-stroke, which is the same as the timeframe in this study. A reason for this domain having the best scores may be because inpatient rehabilitation is centred on functional exercises (Duncan et al. 2003). Edwards et al. (2006) also found that participants scored well with the ADLs but struggled in the other domains. This focus on self-efficacy or ability to complete ADLs in therapy results in a higher ADL score (Pang et al. 2007) but it should be noted that focus on this domain in therapy results in incomplete recovery in the other domains.

Work and education was one of the domains that stroke survivors in this study struggled with the most with a mean score of 2 (SD = 2.3). Participants in a study by Mayo et al. (2002) also struggled most with meaningful activities to fill the day and this included work and education. Vocational rehabilitation post-stroke is not given much attention as stroke usually occurs later in life and this may be a reason why participants in both studies struggled with this domain (Vestling, Tufvesson & Iwarsson 2003). Participants in a study by Edwards et al. (2006) showed decreased satisfaction in their ability to engage in productive pursuits such as work and volunteer activities as well as their ability to travel and participate in leisure and recreational pursuits. This finding is also similar to the one in this study. A reason for low scores with the work and education domain in this study may be that most participants already had low levels of education or were unemployed before they had their stroke and had no intention of going back to school and no work to go back to and no volunteering in community-related projects (unpaid employment).

### Patient satisfaction with physiotherapy services

The mean score for patient satisfaction with physiotherapy services was 92% (SD = 9.17), which indicates that most patients were satisfied with the services. This is more than the 71% of those satisfied with physiotherapy services in a study by Beattie et al. (2002). When patient’s expectations of care are exceeded, levels of satisfaction are high. Patients may view the physiotherapy services offered at CHCs as inferior as there is heavy reliance on hospitals for this acute management while access to community-based rehabilitation facilities is limited (Anderson et al. 2000). These participants may not have expected to encounter physiotherapy services at a community level because of scarcity of this service and this may have led to greater satisfaction because of exceeded anticipated expectations of what the healthcare facility could offer. South Africa has adopted the primary healthcare approach as the most appropriate strategy to meet its healthcare needs and a district health system is in place to meet the healthcare needs of each province with at least one CHC in each of the districts (Rhoda & Hendry 2003). These CHCs provide services that range from preventative and promotive to curative and rehabilitative services, and most of the stroke survivors are seen either once a week or once a month depending on the CHC patient load. The results of a study by Rhoda et al. (2009) revealed that there is a lack of therapy services to provide rehabilitation to survivors of stroke at the CHCs in the Western Cape. The findings in this study also suggest that the amount of time spent on physiotherapy, occupational therapy and speech therapy was low either as a result of the lack of services or an inability to access the CHCs (Rhoda et al. 2009)

Participants’ lowest scores were for accuracy of bills, availability of parking and treatment prices. For the parking item, participants may have viewed this item as the place where their public transport drops them off or where their private taxi parks in relation to the facility because many of the patients who come to these primary healthcare facilities use public transport. Participants may have viewed the treatment prices item as the travel costs they may have incurred because they did not have to pay for services received. This is different to score in a study of patient satisfaction with physiotherapy services by Beattie et al. (2002). Cost of therapy was the one item in their study that had poor scores and this may have been related to the payment patients needed to make for their services.

Patients were happy with the waiting time at the physiotherapy department. The item ‘I was seen promptly when I arrived for treatment’ scored 5 (4 to 5) on the PTPSQ. This could be because of the fact that at the community health centre, a patient is generally given an appointment beforehand and just goes straight to the physiotherapy department upon arrival without having to queue up for the clinic file. This can be viewed as decreased waiting time in comparison to the rest of the clinic.

### Quality of life after stroke

The mean total for the SSQOL for all 108 stroke survivors in this study was 157 out of 245 (SD = 23.16) with the highest score of 235 out of 245 and the lowest score of 54 out of 245. In a study by Ntsiea, Van Aswegen and Lord (2015), the mean total for the SSQOL was 219 with a minimum of 151 and a maximum of 245. Every domain scored higher in the study by Ntsiea et al. (2015) compared to this study. It must be noted that in the study by Ntsiea et al., the SSQOL was assessed up to 6 months after stroke while in this study the SSQOL was assessed up to 4 years after stroke. According to Ahlsiö et al. (1984), ADLs function improved during follow-up assessments, but the QOL did not improve. This explains the findings of this study as QOL seems to have decreased over time.

Participants reported problems with all domains including self-care, social roles and work/productivity. These figures are all lower than the levels found for these domains by Ntsiea et al. (2015), but similar to a study by Hopman and Verner (2003) in which patients reported decline in domains related to their independence, usefulness, self-care and socialising that were as a result of comorbid conditions, reduced energy levels, limited social life and unrealistic expectations of recovery after stroke.

Mean scores for social roles (14 out of 20) and work and productivity (9 out of 15) were also poor. These scores were lower than those found in the study by Ntsiea et al. (2015), which is an indication that most participants in this study did not have meaningful activity, which includes returning to work or school post-stroke. Returning to work after stroke has a positive impact on QOL (Ntsiea et al. 2015) and looking at the high levels of unemployment in this study, this statement holds true as the QOL of the stroke survivors in this study was poor especially in the work and productivity domain.

### Caregiver strain

Most of the caregivers (55%) in this study were strained. The percentage in this study is much lower than the caregiver strain measured in the studies by Mudzi (2010) and Hilton et al. (2013), which were 90% and 77.1%, respectively. Their studies focused more on participants who required a caregiver for core ADLs, whereas participants included in this study did not need to have a caregiver and thus the patients’ level of functional dependence may be less in this study leading to relatively less number of strained caregivers. The percentage of strained caregivers is relatively lower in the study by Blake et al. (2003) in which 40% of the caregivers were found to be strained after 6 months. This study was conducted in middle to high socioeconomic settings, which could mean that the caregivers’ socioeconomic conditions may be relatively better resulting in less strain. This study and those by Mudzi (2010) and Hilton et al. (2013) were done in low socioeconomic settings. People living in these settings may already have had some form of financial or physical strain prior to the stroke. Thus, stroke in addition to the pre-existing strain may have predisposed the caregivers to additional strain.

### Relationship between community reintegration and Caregiver strain index, stroke survivors’ quality of life and patients’ satisfaction with physiotherapy services

There was a positive correlation between MSCRIM and SSQOL, which shows that as community reintegration improves, so does the survivor of strokes’ QOL. QOL is affected by levels of physical impairment, which affects functional outcomes and community reintegration (Carod-Artal et al. 2000) and another factor that negatively affects community reintegration is the inability to complete ADLs (Pang et al. 2007). Participants in this study had few problems with their ADLs and most of them had reintegrated well into their communities. Thus, it makes sense that there is a positive association between MSCRIM and SSQOL in this study.

There was a weak positive relationship between MSCRIM and PTPSQ showing a higher level of satisfaction with physiotherapy services in participants with higher levels of community reintegration. Pound et al. (1994) stated that better levels of integration did not necessarily lead to high levels of satisfaction, but led to better adherence to exercise programmes (Hush et al. 2011). This may have meant the participant identified with or trusted the healthcare facility more and this would have led to higher levels of exercise adherence, which would improve their functional ability and eventually lead to better community reintegration.

There was a negative correlation between community reintegration and levels of caregiver strain. Hillier and Inglis-Jassiem (2010) suggests that patients who return home with lower levels of reintegration are more of a physical and financial burden on their family and this holds true in this study as this study showed physical strain and financial strain as factors that contributed to caregiver’s levels of strain. Personal and behavioural changes in the participants were reported as reasons for caregiver strain and this is similar to the findings by Mudzi (2010). Thus, it makes sense that community reintegration is negatively correlated to caregiver strain in this study.

### Limitations of the study

Lack of clarity regarding costs of therapy as well as understanding of what was meant by the question related to parking in the PTPSQ may have made the results of the PTPSQ less accurate.

## Conclusion

This study’s findings are similar to what is in the literature in that not all stroke survivors are reintegrated into their community and that most of them have poor QOL. This decreased level of reintegration leads to increased levels of caregiver strain. Although the stroke survivors may not have fully reintegrated into the community, they did experience high levels of satisfaction with the physiotherapy service.
